# Clinical Experience of Using Remimazolam Instead of Volatile Anesthesia for Near-Infrared Photoimmunotherapy

**DOI:** 10.7759/cureus.88128

**Published:** 2025-07-16

**Authors:** Junya Matsumi, Tetsufumi Sato

**Affiliations:** 1 Department of Anesthesiology and Intensive Care Medicine, National Cancer Center Hospital, Tokyo, JPN

**Keywords:** blood pressure (bp), general anesthesia, heart failure with reduced ejection fraction, mean arterial pressure (map), midazolam, remimazolam, sevoflurane

## Abstract

Remimazolam is a newly-approved anesthetic agent, characterized by minimal hemodynamic fluctuations. We present a case of a patient who received three courses of near-infrared photoimmunotherapy for nasopharyngeal cancer. The patient had heart failure with reduced ejection fraction and aortic valve regurgitation. During the first two courses, the patient was anesthetized by sevoflurane with induction of midazolam and opioids and suffered hypotension requiring continuous infusion of noradrenaline. In the third course, the patient was anesthetized with remimazolam and opioids, and showed hemodynamic stability without the use of noradrenaline. This case report suggests the usefulness of remimazolam for hemodynamically vulnerable patients.

## Introduction

Despite careful management, hemodynamic instability such as hypotension often occurs during general anesthesia. Intraoperative hypotension is a risk factor for anesthesia-related serious adverse events [1].

Remimazolam is an ultra-short-acting benzodiazepine and has recently been approved as a general anesthetic agent. It is characterized by minimal hemodynamic fluctuations [2,3]. Due to this, remimazolam is often used for patients with cardiovascular diseases. However, no study compares remimazolam and different anesthetic agents in the same patient during the same surgical procedure over several months. 

Near-infrared photoimmunotherapy (NIP) is a newly developed cancer therapy. In Japan, patients with unresectable locally advanced or locally recurrent head and neck cancers are eligible for multiple NIP sessions [4]. NIP is a photodynamic therapy, and combines the specificity of intravenously-injected antibodies with the destructive power of near-infrared light to selectively kill cancer cells while minimizing damage to healthy tissue. NIP can reach deep tissue and give rise to more photochemical reactions than other photodynamic therapies. One of the most severe side effects of photoimmunotherapy (PIT) is pain. In a phase I trial with this therapy, 100% of the patients reported pain at the application site, and pain of Grade 3 or above was observed in 33.3% of patients [5]. Due to severe pain, general anesthesia is necessary for NIP. Since alcohol consumption and smoking, which are risk factors for head and neck cancers, are also risk factors for cardiovascular diseases, many patients with head and neck cancer also have cardiovascular diseases [6,7]. Therefore, many patients undergoing NIP are thought to be susceptible to hemodynamic instability caused by general anesthesia.

This case report demonstrates the usefulness of remimazolam-total intravenous anesthesia (TIVA) for a patient with cardiovascular disease by using different anesthetic agents during the same surgical procedure over several months. This manuscript adheres to the CAse REport or CARE guidelines [8].

## Case presentation

A 75-year-old male patient (height: 167.4cm and weight: 52.2 kg) had a history of esophageal cancer with complete remission by chemoradiotherapy. Also, the patient had an oropharyngeal cancer with good partial response by chemotherapy after transoral videolaryngoscopic surgery (TOVS) and resection of a metastatic lung tumor five years ago. A year ago, the patient underwent TOVS for nasopharyngeal cancer. Then, the patient underwent total pharyngolarygoesophagectomy (TPL) for a radical cure four months ago. However, recurrence of nasopharyngeal cancer was detected. Then, NIP was considered as a treatment for this recurrence of nasopharyngeal cancer.

The patient also had cardiovascular comorbidities. Two years ago, he developed congestive heart failure. During that examination, myocardial infarction was detected, and the patient underwent percutaneous coronary intervention in the left anterior descending artery. Subsequently, he developed chronic heart failure with reduced ejection fraction. His preoperative echocardiography showed left ventricle dysfunction (antero-septal wall motion akinesis and ejection fraction of 28% by modified Simpson method), ventricular aneurysm, and valvular heart diseases (moderate aortic valve regurgitation and mild mitral valve regurgitation (video 1)).

**Video 1:** Preoperative echocardiography Preoperative echocardiography showed left ventricle dysfunction (antero-septal wall motion akinesis and ejection fraction of 28% by modified Simpson method), ventricular aneurysm, and valvular heart diseases (moderate aortic valve regurgitation and mild mitral valve regurgitation).

This heart failure was compensated based on exercise tolerance, and the patient was assessed to be tolerable for surgery. 

Then, the patient was scheduled for three courses of NIP. In our hospital, pulse oximeter, capnometer, ventilatory volume monitor, electrocardiogram, non-invasive blood pressure, temperature, and neuromuscular monitor are used in all general anesthesia. And as this patient had cardiovascular risk, an invasive arterial monitor was used during anesthesia for all the three course of NIP.

**Figure 1:**
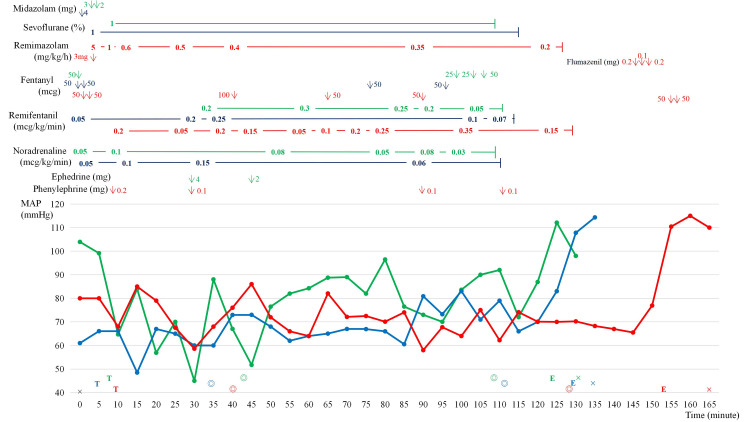
The trends of mean arterial pressure (MAP) and the usage of drugs during the three courses of near-infrared photoimmunotherapy (NIP) ×: the start and end of anesthesia; ◎: start and end of surgery; T: tracheal intubation; E: extubation The green line and the symbols show the trend of MAP and anesthetic management during the first course. The blue line and associated symbols show the trend of MAP and anesthetic management during the second course. For the maintenance of adequate MAP, continuous infusions of noradrenaline (Nad) were required in these two courses. The red line, along with the symbols, shows the trend of MAP and anesthetic management during the third course. In the third course, MAP was maintained without using Nad. Hemodynamics were comparable across the three courses of anesthetic management.

For TPL, the patient was anesthetized with sevoflurane and remifentanil. Intraoperative hemodynamics were acceptable along with a continuous infusion of noradrenaline (Nad). Therefore, the same anesthetic was planned for the first course of NIP. In the first course of NIP, although intraoperative hemodynamics were stable under continuous infusion of Nad, hypotension necessitating continuous infusion of Nad was observed between induction with midazolam and the opioid (fentanyl 50 mcg) and the start of surgery (green line in Figure 1). 

During the first course, operation and anesthesia times were 62 minutes and 130 minutes, respectively, and intraoperative fluid balance was 50 mL. In this anesthesia, the concentration of sevoflurane was titrated by electroencephalogram (EEG) and Bispectal Index (BIS) around 50. In the second course of NIP at four months after the first course, although intraoperative hemodynamics were stable under continuous infusion of Nad, hypotension necessitating continuous infusion of Nad was observed again between induction with midazolam and opioid (fentanyl 100 mcg with remifentanil 0.05 mcg/kg/min) and the start of surgery (blue line in Figure 1). During the second course, operation and anesthesia times were 75 minutes and 135 minutes, respectively. And intraoperative fluid balance was 750 mL. During this course, the depth of anesthesia was monitored using the end-tidal concentration of sevoflurane, but EEG was not monitored.

Due to repeated hypotension necessitating continuous infusion of Nad under careful anesthesia management, remimazolam-TIVA was selected in the third course of NIP at four months after the second course. Anesthesia induction was started at remimazolam 5 mg/kg/h continuous infusion after 3 mg bolus infusion with fentanyl 100 mcg. Just after tracheal intubation from permanent tracheostoma, blood pressure decreased. However this hypotension was immediately recovered by a single administration of phenylephrine (0.2 mg). After a temporary decrease, intraoperative hemodynamics remained stable without use of Nad during anesthesia, titrated by BIS between 45 to 60. During the third course, operation and anesthesia times were 97 minutes and 164 minutes, respectively, and intraoperative fluid balance was 455 mL. As a result, mean arterial pressure was maintained without continuous infusion of Nad (red line in Figure 1).

At one month after the third course of NIP, the therapeutic effect was considered progressive disease. As his general condition was judged to be tolerable, the chemotherapy using pembrolizumab was started. And, its outpatient chemotherapy continues.

## Discussion

This case report describes the management of a hemodynamically vulnerable patient during remimazolam-TIVA. In this case, the patient exhibited hypotension during anesthesia with midazolam and sevoflurane during the same surgical procedure. The total time of hypotension (defined as mean arterial pressure under 60 mmHg) during anesthesia was comparable across the three courses of NIP (Table 1).

**Table 1:** Total duration of hypotension, fluid balance, and total amount of drugs at every courses Total duration of hypotension, defined as mean arterial pressure below 60 mmHg, was comparable among the three courses. Also, the total amount of opioids given during the third course was similar to the previous two courses.

	First course	Second course	Third course
Total duration of hypotension (min)	11 (8.5% of anesthesia time)	12 (8.9% of anesthesia time)	13 (7.9% of anesthesia time)
Anesthesia time (min)	130	135	164
Noradrenaline (mg)	0.45	0.64	0
Ephedrine (mg)	6	0	0
Phenylephrine (mg)	0	0	0.5
Intraoperative fluid balance (mL)	50	750	455
Remifentanil (mg)	0.83	1.13	1.38
Fentanyl (mg)	0.15	0.2	0.4

Although the depth and pain management of remimazolam-TIVA was not light, based on opioid doses and BIS value, there was no need to administer Nad during the third course. Since the administration of Nad carries a risk of tissue necrosis, its administration requires a central venous tract. During head and neck surgery, more attention is paid as the supine position, with the upper extremity aligned with the body trunk, is often used. As sterile cloth is usually covering the body of patients, it is difficult to check the entry point of peripheral venous tract at this intraoperative position. Therefore, it takes time to notice any leak in the peripheral venous tract. And, this intraoperative position is also used at NIP. Thus, the risk of tissue necrosis for Nad could be serious.

One of the advantages of remimazolam is hemodynamic stability [2]. There are limited data about remimazolam pharmacodynamics, but it is structurally similar to midazolam. Therefore, remimazolam pharmacodynamics are broadly similar to midazolam [9]. Midazolam usually shows a small increase in heart rate and a decrease in systemic vascular resistance. The reduction of preload and afterload may improve cardiac output, especially in the case of cardiac dysfunction [9]. Hemodynamic change occurs both during induction and maintenance of anesthesia. Many previous studies show the superiority of remimazolam-TIVA compared to propofol-TIVA [3,9-12]. In comparison to the previous studies, midazolam was used at induction in the first two courses of this case. Midazolam is considered to cause mild hemodynamic fluctuation [13]. As these drugs belong the same group, the impact on hemodynamics might be similar for midazolam and remimazolam.

However, hypotension around induction was observed in this case. One possible explanation is the transition of drugs from midazolam to sevoflurane due to the change in status from induction to maintenance of anesthesia. In fact, some studies reported greater hemodynamic stability during the maintenance of anesthesia using remimazolam than using sevoflurane [14,15]. While few studies have evaluated anesthesia with remimazolam-TIVA compared to sevoflurane with midazolam induction, this case report showed the superiority of remimazolam-TIVA compared to both induction with midazolam and maintenance with sevoflurane. In addition, the adequate dose of remimazolam has not been established. In this case, remimazolam was administrated at about 0.05 mg/kg bolus and continuous administration of 5 mg/kg/h. This dose during induction was set based on a previous study [16]. After induction, remimazolam was titrated by BIS, and this dose of remimazolam was considered appropriate in this patient as per BIS value.

Another feature of this case report is the situation. Although the type of surgery is often identical, it is not common to use different interventions on the same patient in clinical studies. Moreover, a crossover study at the induction of anesthesia is methodically difficult. Therefore, this case report, which evaluated different anesthetics for the same procedure in the same patient over several months, is valuable. Hemodynamics during anesthesia is affected by the depth of anesthesia and pain management. In this case, the depths of anesthesia assessed by BIS or end-tidal concentration were comparable. Also, the dose of opioids during induction may affect hemodynamic instability. As the amount of opioid in the third course was not less than that in the first and the second courses, it is difficult to believe that inadequate pain management was the cause of hemodynamic stability in the third course [16]. Since the amount of opioid at third course was comparable to the previous two courses, we believe the anesthetics were the main factor affecting hemodynamics.

There are several limitations to this case report. First, as remimazolam-TIVA was administered only once, the reproducibility of its hemodynamic stability is unknown. Second, hemodynamics was assessed by blood pressure alone. However, blood pressure is the most frequently used parameter in the clinical setting and chosen as the endpoint for hemodynamics in many studies. Therefore, we believe that this limitation might be acceptable [5-7,9,11]. Third, BIS monitoring was not used during the second course of NIP. However, the depth of sedation was monitored by the end-tidal concentration, which is the standard monitoring method for volatile anesthesia. Also, the concentration of sevoflurane during the second course of NIP was similar to the first course. Therefore, we assessed that the depth of sedation was comparable. Lastly, the selection and use of drugs are not protocolized and determined by every anesthesiologist. Instead of protocol, case conferences are held for all patients in our department. In this case, hemodynamic depression occurred and the continuous infusion of Nad was effective for hemodynamic stability in the management of TPL. As a result of the case conference, Nad was administrated from the beginning in the previous two courses. These case conferences make it possible for us to offer consistent anesthetic management.

## Conclusions

In this case report, the patient received both remimazolam anesthesia and midazolam-sevoflurane anesthesia during three courses of the same surgical procedure. And, hemodynamics in remimazolam anesthesia without Nad infusion was comparable to that in midazolam-sevoflurane anesthesia with Nad infusion.

It reaffirms that remimazolam anesthesia can be a viable option for patients with unstable hemodynamics. However, further research is needed to identify patients and the surgical types in which remimazolam-TIVA is needed. Also, it is expected that studies will be conducted to evaluate the appropriate dose of remimazolam at induction for patients with unstable hemodynamics.

## References

[1] Walsh M, Devereaux PJ, Garg AX (2013). Relationship between intraoperative mean arterial pressure and clinical outcomes after noncardiac surgery: toward an empirical definition of hypotension. Anesthesiology.

[2] Sneyd JR, Gambus PL, Rigby-Jones AE (2021). Current status of perioperative hypnotics, role of benzodiazepines, and the case for remimazolam: a narrative review. Br J Anaesth.

[3] Zhang H, Li H, Zhao S, Bao F (2024). Remimazolam in in general anesthesia: a comprehensive review of its applications and clinical efficacy. Drug Des Devel Ther.

[4] Yamada M, Matsuoka K, Sato M, Sato K (2023). Recent advances in localized immunomodulation technology: application of NIR-PIT toward clinical control of the local immune system. Pharmaceutics.

[5] Tahara M, Okano S, Enokida T (2021). A phase I, single-center, open-label study of RM-1929 photoimmunotherapy in Japanese patients with recurrent head and neck squamous cell carcinoma. Int J Clin Oncol.

[6] Verro B, Saraniti G, Fiumara S, Ottoveggio G, Saraniti C (2024). Smoking and alcohol habits in head and neck cancers: how many patients stop after diagnosis?. J Cancer Policy.

[7] Ohlrogge AH, Frost L, Schnabel RB (2022). Harmful impact of tobacco smoking and alcohol consumption on the atrial myocardium. Cells.

[8] Gagnier JJ, Kienle G, Altman DG, Moher D, Sox H, Riley D (2013). The CARE guidelines: consensus-based clinical case reporting guideline development. J Med Case Rep.

[9] Dundee JW, Halliday NJ, Harper KW, Brogden RN (1984). Midazolam. A review of its pharmacological properties and therapeutic use. Drugs.

[10] Dai G, Pei L, Duan F (2021). Safety and efficacy of remimazolam compared with propofol in induction of general anesthesia. Minerva Anestesiol.

[11] Liu T, Lai T, Chen J, Lu Y, He F, Chen Y, Xie Y (2021). Effect of remimazolam induction on hemodynamics in patients undergoing valve replacement surgery: a randomized, double-blind, controlled trial. Pharmacol Res Perspect.

[12] Ju JW, Lee DJ, Chung J, Lee S, Cho YJ, Jeon Y, Nam K (2024). Effect of remimazolam versus propofol on hypotension after anesthetic induction in patients undergoing coronary artery bypass grafting: a randomized controlled trial. J Clin Anesth.

[13] Lee B, Kim MH, Kong HJ, Shin HJ, Yang S, Kim NY, Chae D (2022). Effects of remimazolam vs. sevoflurane anesthesia on intraoperative hemodynamics in patients with gastric cancer undergoing robotic gastrectomy: a propensity score-matched analysis. J Clin Med.

[14] Ko E, Je LG, Kim JH, Song YJ, Lim CH (2024). Effects of remimazolam versus sevoflurane on hemodynamics in patients undergoing coil embolization of cerebral aneurysm: a prospective randomized controlled trial. J Clin Med.

[15] Miyoshi H, Watanabe T, Kido K (2022). Remimazolam requires less vasopressor support during induction and maintenance of general anesthesia in patients with Severe aortic stenosis undergoing transcatheter aortic valve replacement: a retrospective analysis from a single center. Biomed Res Int.

[16] Teixeira MT, Brinkman NJ, Pasternak JJ, Abcejo AS (2024). The role of remimazolam in neurosurgery and in patients with neurological diseases: a narrative review. J Neurosurg Anesthesiol.

[17] Sawano Y, Miyazaki M, Shimada H, Kadoi Y (2013). Optimal fentanyl dosage for attenuating systemic hemodynamic changes, hormone release and cardiac output changes during the induction of anesthesia in patients with and without hypertension: a prospective, randomized, double-blinded study. J Anesth.

